# Feed gas effect on plasma inactivation mechanism of *Salmonella* Typhimurium in onion and quality assessment of the treated sample

**DOI:** 10.1038/s41598-017-17579-5

**Published:** 2017-12-18

**Authors:** Muhammad Saiful Islam Khan, Eun-Jung Lee, Seok-In Hong, Yun-Ji Kim

**Affiliations:** 1 Department of Food Biotechnology, University of Science and Technology, Daejeon, 305-350 Republic of Korea; 2 Faculty of Food Science and Biotechnology, College of Life Science, Sejong University, 209 Neungdong-ro, Gwangjin-gu, Seoul 05006 Republic of Korea; 3 Food Safety Research Group, Korea Food Research Institute, 245 Nongsaengmyeong-Ro Iseo-Myeon, Wanju-Gun, Jeollabuk-Do 55365, Republic of Korea

## Abstract

A submerged dielectric barrier discharge (DBD) plasma reactor was used to inactivate artificially inoculated reference strains of *Salmonella* Typhimurium ATCC 14028 on sliced onion (3 cm × 3 cm). *Salmonella* Typhimurium reductions obtained after 10 min of treatment were 3.96 log CFU/slice and 1.64 log CFU/slice for clean dry air and N_2_ feed gas, respectively. Variations observed in Optical Emission Spectra (OES) for different feed gases are responsible for the inactivation level variations of *Salmonella* Typhimurium. The physiochemical properties of the onion slices, such as quercetin content, ascorbic acid content and color parameters, were monitored before and after treatment and the changes that occurred were measured to be in the acceptable range. Quercetin content was reduced only 3.74–5.07% for 10 min treatment, higher reduction was obtained for the use of clean dry air than that of N_2_ feed gas. Ascorbic acid loss was measured to be 11.82% and 7.98% for a 10 min treatment with clean dry air and N_2_ feed gas, respectively. The color parameters did not show significant changes upon treatment (p > 0.05) of the same duration for the uses of different feed gases.

## Introduction

In our daily diet, fresh vegetables and fruits are essential items. The application of certain processing technologies and widespread use of pesticides to keep foods fresh and safe are becoming growing concerns in the context of food safety issues^1^. Bacterial contamination accounts for 34% of all global food safety issues every year^2^. The recent *Escherichia coli* O157:H7 contaminations of cucumbers in Europe and Listeria-tainted cantaloupe in the US have raised renewed awareness regarding food safety across the world^3^. Therefore, effective and easy-to-apply approaches for pathogen inactivation in fruits and vegetables have taken on a high priority. Several thermal, chemical and non-thermal sterilization methods, such as irradiation by ultraviolet (UV) and gamma rays, have been used to eliminate microbes. For instance, while thermal treatment is effective because of its high temperature (approximately 121 °C or 134 °C), it is not suitable for use in food processing because of its negative effects on food quality with respect to nutrient standards and sensory properties and due to its high energy use^4^. Chemical treatments, such as chlorinated water, formaldehyde, ethylene oxide (EtO), H_2_O_2_ and other chemical compounds for sterilization, are of limited applicability because of their toxicity^5^. In the case of UV and gamma ray irradiation, the generated energetic photons can seriously damage the structure of DNA and, most importantly, such photons are carcinogenic in humans^6^. The above mentioned drawbacks are the major causes of the large scale practical utilization.

Thus, a variety of research efforts are ongoing worldwide to develop novel techniques that can effectively eliminate microbial contaminants without degrading the sensory quality and functional properties of treated foods^7^. Among the non-thermal inactivation techniques, increased attention has been devoted to physical inactivation methods, such as the plasma inactivation technique^8^. The use of thermal plasma is limited due to its high temperature (2,000–10,000 K) as elevated temperature causes the tissue damage^9^. On the other hand, the non-thermal plasma method works at room temperature, and due to its high efficiency and safety, it has been demonstrated to be appropriate for various applications such as surface modification of polymers^10^, air purification^11^, and sterilization for biological and medical purposes^12^. Dielectric Barrier Discharged (DBD) plasma under water is a fast and reliable non-thermal plasma technique that has been used extensively for microbe inactivation^13^. Environmentally friendly plasma discharged water has been widely studied in the context of environmental and wastewater treatment^14^, and it has recently been introduced in bacterial inactivation studies for food safety^15^. A number of DBD plasma discharge techniques have been introduced to inactivate bacteria, and different reduction levels for *Escherichia coli* O157:H7 were obtained using different discharges and devices^15^. Various reactive species were reported to give rise to different levels of inactivation of *Escherichia coli* O157:H7. Different types of reactive species are generated by different types of electrical discharge; for example, pressure waves and UV light are generated by pulsed high-current discharge underwater, low pH and hydrogen peroxide (H_2_O_2_) are generated by gliding arc discharge, and atomic oxygen, atomic hydrogen and hydroxyl (OH) radical species are generated by capillary discharge. Dielectric barrier discharge (DBD) produces ozone, UV light, reactive oxygen species (ROS) and reactive nitrogen species (RNS), which exhibit high bacterial inactivation capability^16^. Additionally, variations in gas composition can give rise to differences in the inactivation of food-borne pathogens because different gas components can produce different types of reactive species^17^. Air, oxygen, nitrogen, argon, and helium are commonly used as feed gases for plasma generation. Because argon and helium are expensive, both air and nitrogen may have greater potential for industrial applications. Although air gas has often been used to generate plasma^18^, nitrogen gas has been used as often as air in studies of bacterial pathogen inactivation^19^. The complete elimination of the microorganisms depends on several factors such as the plasma power, the gas, the type of bacteria, and the type of medium.

Onion (*Allium cepa* L.) is one of the most commonly consumed vegetables^20^. It is recognized for its various biological activities, such as antioxidant and antibacterial effects, which are mediated by the presence of sulfur, phenolic, and selenium compounds^21^. The reactive radicals and other active species present in DBD plasma discharged water play key roles in microbial inactivation processes; on the other hand, due to the short lifetimes of radicals, they are unable to leave any residual trace contaminants behind^22^. Therefore, DBD plasma discharged water may change the chemical contents of the treated onions. Among the chemical contents, quercetin and ascorbic acid are at high risk due to their OH radical scavenging capacity^23^. Quercetin is one of the flavonoids present in onion with a wide range of health benefits, including antioxidant, antithrombotic, anti-asthmatic and antibiotic effects^21^. Vitamins are important component of fresh fruit and vegetables, and among them, vitamin C, which is mainly ascorbic acid, is the most common ingredient^24^. However, until now the physiochemical quality changes of treated vegetables have not been studied extensively. Among the physical parameters, color is probably the primary quality factor for the consumer’s acceptance. Hence, it becomes imperative to evaluate the changes in the physiochemical properties of onions before and after treatment with DBD plasma discharged water. In this study, we investigated the inactivation efficacy and mechanism for different feed gases used as well as the physiochemical quality changes of onion caused by DBD plasma discharged water treatment. DBD plasma discharged water was applied to slices of onions contaminated with *Salmonella* Typhimurium, which is the most common cause of infections leading to nausea, vomit, fever, diarrhea or even death. The inactivation efficacy was evaluated by counting the colony forming units (CFU) before and after plasma treatment. The quercetin and ascorbic acid (Vitamin C) content of the onion slices was monitored quantitatively by HPLC before and after plasma treatment. The color of the slices was also evaluated by a colorimeter to obtain precise values for the color change.

## Results

### Radicals produced by the DBD plasma discharged water

**Figure 1:**
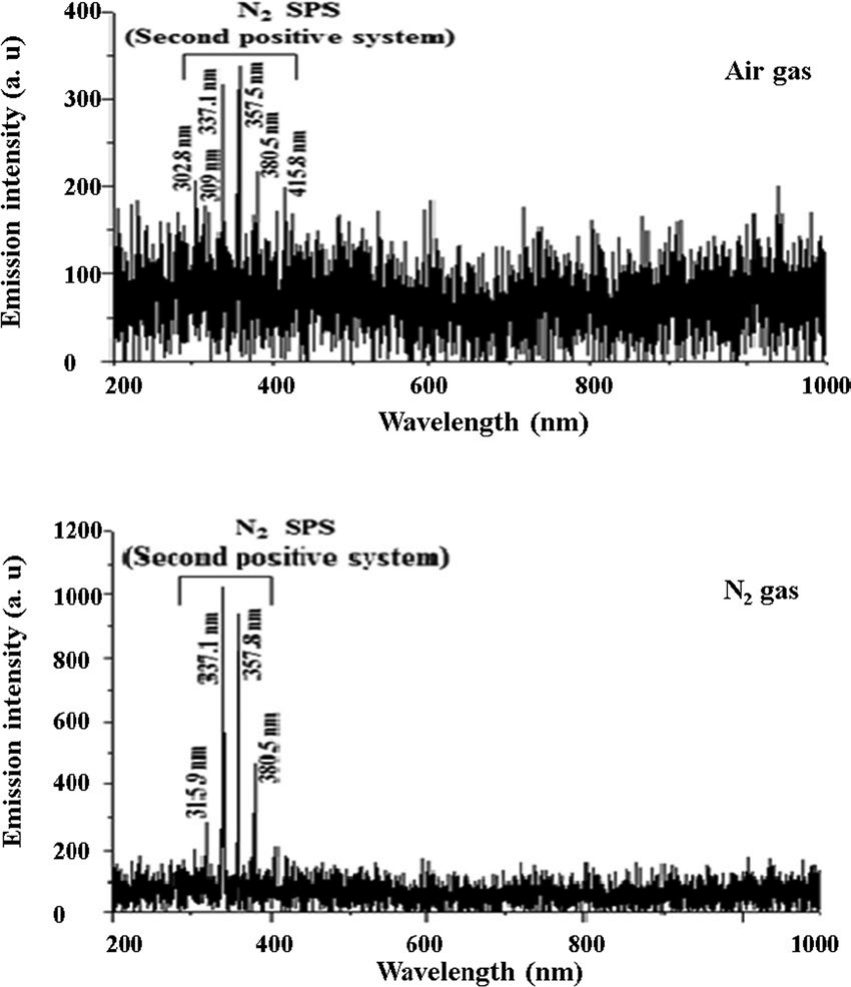
Optical emission spectra (OES) of clean dry air and N_2_ gas plasma generated from a dielectric barrier discharge plasma reactor.

Optical emission spectroscopy (OES) of the DBD plasma discharge used in this experiment shows that OH radicals and the 2^nd^ positive system N_2_ (C-B) gave rise to the main peaks in the near-UV region (300–400 nm) of the OES spectrum in Fig. 1 for clean dry air gas. These peaks imply that OH radicals and RNS were produced by the DBD plasma discharge. Ozone gas (O_3_) and hydrogen peroxide (H_2_O_2_) were also generated in the water by DBD plasma discharge^25^. N_2_ feed gas also shows that the 2^nd^ positive system N_2_ (C-B) gave rise to the main peaks in the near-UV region (300–400 nm) of the OES spectrum, except peak 309 (OH), and this could be the reason for the partial inertness of N_2_ plasmas. In our previous study^25^, it was observed that after 6, 4 and 8 min of plasma treatment time the amount generated for OH radical was 1.81 × 10^−5^ M, dissolved ozone gas was 1.5 ppm and H_2_O_2_ was 2.5 × 10^−6^ M, respectively. The data for dissolved ozone and H_2_O_2_ until 8 and 10 min, respectively, showed a constant trend. In the case of the OH radical, it was observed that after 6–8 min of DBD operation, the intensity or concentration of 2-hydroxyterephthalic acid (HTA) or OH radical (1 mol of OH radical ≡ 1 mol of HTA) was decreased whereas the concentration of HTA (OH radical) was supposed to either increase or remain constant. The phenomenon of decreasing OH radical was described in detail with experimental evidence in previous work^25,26^. Mutual reactions occurring among the species generated might be the cause of all three species remaining constant throughout the plasma treatment. Detailed data were not included in this manuscript as no significant difference from previous work was observed regarding the final concentration of all the species.

### Salmonella Typhimurium inactivation by DBD plasma discharged water

**Figure 2:**
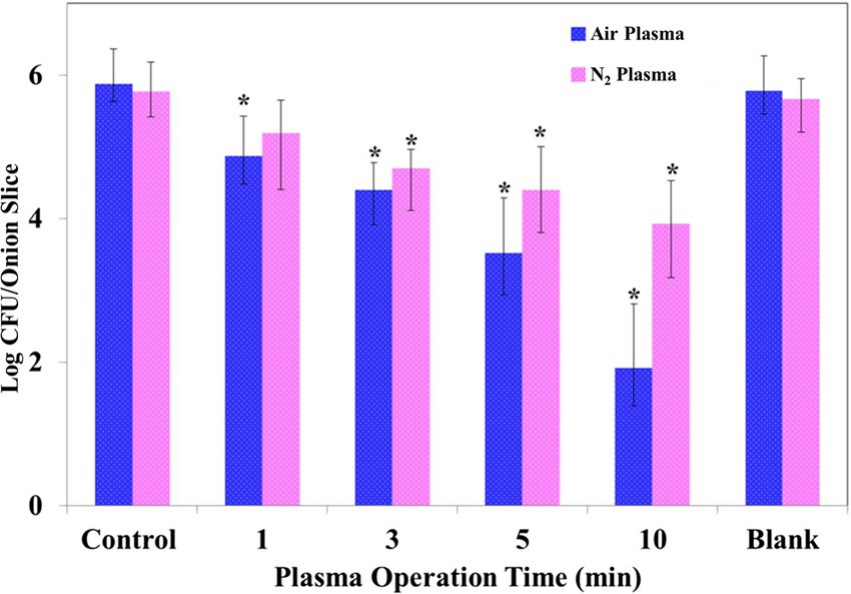
Inactivation of *Salmonella* Typhimurium ATCC 14028 on onion slices by BDB plasma discharged water with air and N_2_ feed gases. *Denotes significant difference compared with untreated control, Mann–Whitney-U test (p < 0.05).

DBD plasma discharged water was applied to reduce *S*. Typhimurium inoculated on sliced onion (3 cm × 3 cm). The onion slices were placed in a polypropylene jar containing 2.0 L of distilled water. To generate plasma two different feed gases i. e., clean dry air gas and pure N_2_ gas was blown separately and the inactivation effect for two different feed gases were evaluated. The reduction in inoculated *S*. Typhimurium was 3.96 log CFU/slice onion and 1.64 log CFU/slice onion after 10 min of DBD plasma treatment inside water by clean dry air and N_2_ gas, respectively. Samples were collected at 1, 3, 5 and 10 min following the start of plasma discharge. Figure 2 shows the viability of *S*. Typhimurium determined with respect to the DBD plasma treatment time, with the number of survivors observed as colony-forming units (CFU). Result from blank experiment confirms that the water circulation decreases the viable count by approximately 4%; the inactivation difference observed for 10 min of water flow was much lesser than the inactivation observed in association with plasma operation (Fig. 2). This observation suggests that cell detachment occurs because of the plasma treatment and not because of water circulation.

### Quercetin content evaluation

**Figure 3:**
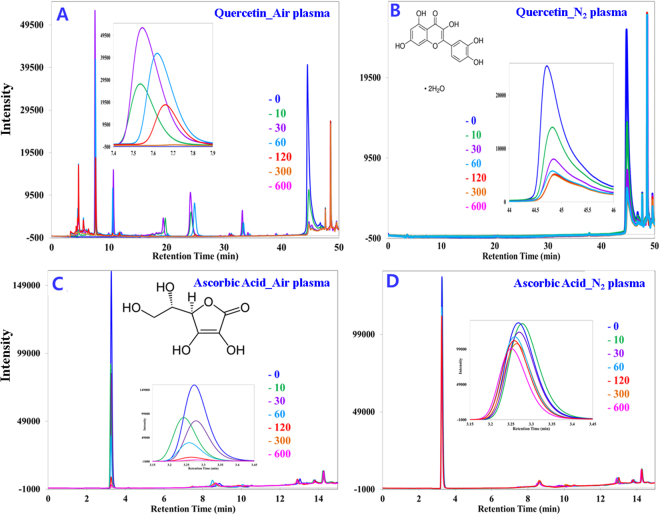
HPLC chromatograms (monitored at 280 nm) showing the reduction of the quercetin and ascorbic acid standard and fragmented smaller molecular weight products for different plasma operation times. (**A**) The quercetin standard treated with clean dry air feed gas. Inset: The fragmented product obtained at retention time 7.4 to 7.9 min. (**B**) The quercetin standard treated with N_2_ feed gas. Inset: Quercetin obtained at retention time approx. 45 min. (**C**) The ascorbic acid standard treated with clean dry air feed gas. Inset: ascorbic acid obtained at retention time approx. 3 min. (**D**) The ascorbic acid standard treated with N_2_ feed gas. Inset: ascorbic acid obtained at retention approx. 3 min.

**Figure 4:**
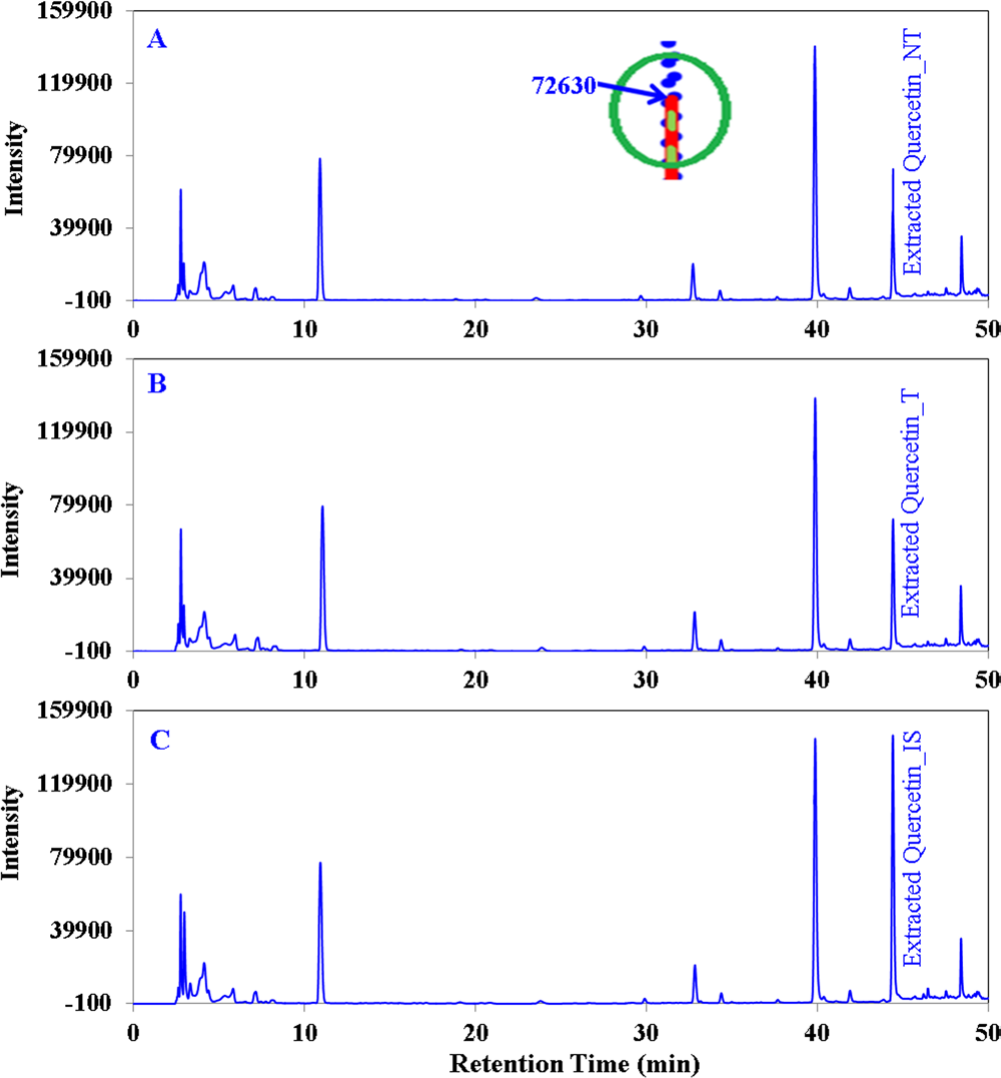
HPLC chromatograms (monitored at 280 nm) showing quercetin extracted from onion slices. (**A**) Non-treated, (**B**) treated with air (**C**) with internal standard to ensure accurate identification of quercetin. Inset: magnified superimposed peak view of three chromatograms at intensity 72630 to assess the reduction level of quercetin easily.

Figure 3A shows that the quercetin standard was completely destroyed within 5 min of plasma operation with clean dry air feed gas. Several fragmented compounds were observed at different retention times (4.67, 7.55, 10.76, 24.13, 33.19 min). The fragmented compounds show an increasing and decreasing pattern in the chromatogram peak observed at different retention times. For example, most of the peaks increase until 60 to 90 sec of treatment and then decline; for instance a magnified view for the product obtained at 7.5 min is shown as an inset in Fig. 3A. In the case of N_2_ feed gas, 95% of the quercetin standard was destroyed within 10 min of plasma operation (Fig. 3B), and no fragmented compounds were identified by our present setup; a magnified view of the quercetin standard obtained at 44 min is shown as an inset in Fig. 3B. A reduction of only 3.74–5.01% (Table 1) of the quercetin content of onion slices occurs after plasma treatment; clean dry air gas treatment shows a greater reduction compared to the treatment with nitrogen gas. Figure 4A and B show chromatograms of extracted quercetin before and after plasma treatment with clean dry air gas. Figure 4C shows the spectrum with the addition of an internal standard to ensure that the extracted product is the target analyte. The chromatograms obtained before and after treatment are similar in terms of the peak obtained at different retention times. Blank experimental result confirms that 2.13% quercetin reduction occurs due to leaching of water for the highest operation time (10 min) used in this experiment. No significant differences were observed for the alteration of the feed gas used.

**Table 1:** Determination of the loss of Quercetin and Ascorbic Acid content for the 10 min of plasma treatment for two different feed gases used in this study.

Feed gas	Component	Before Plasma treatment (mg/100 g, Mean)	After Plasma treatment (mg/100 g, Mean)	Loss (%), Mean	Loss due to leaching (%), Mean	Loss contributed by plasma only (%), Mean ± SD
Air	Quercetin	29.15	27.68	5.07	2.13	2.94 ± 0.27
	Ascorbic Acid	3.40	2.93	11.82	5.21	6.61 ± 0.52
Nitrogen	Quercetin	31.05	29.89	3.74	2.17	1.61 ± 0.21
	Ascorbic Acid	3.51	3.23	7.98	5.01	2.77 ± 0.70

### Ascorbic acid content evaluation

**Figure 5:**
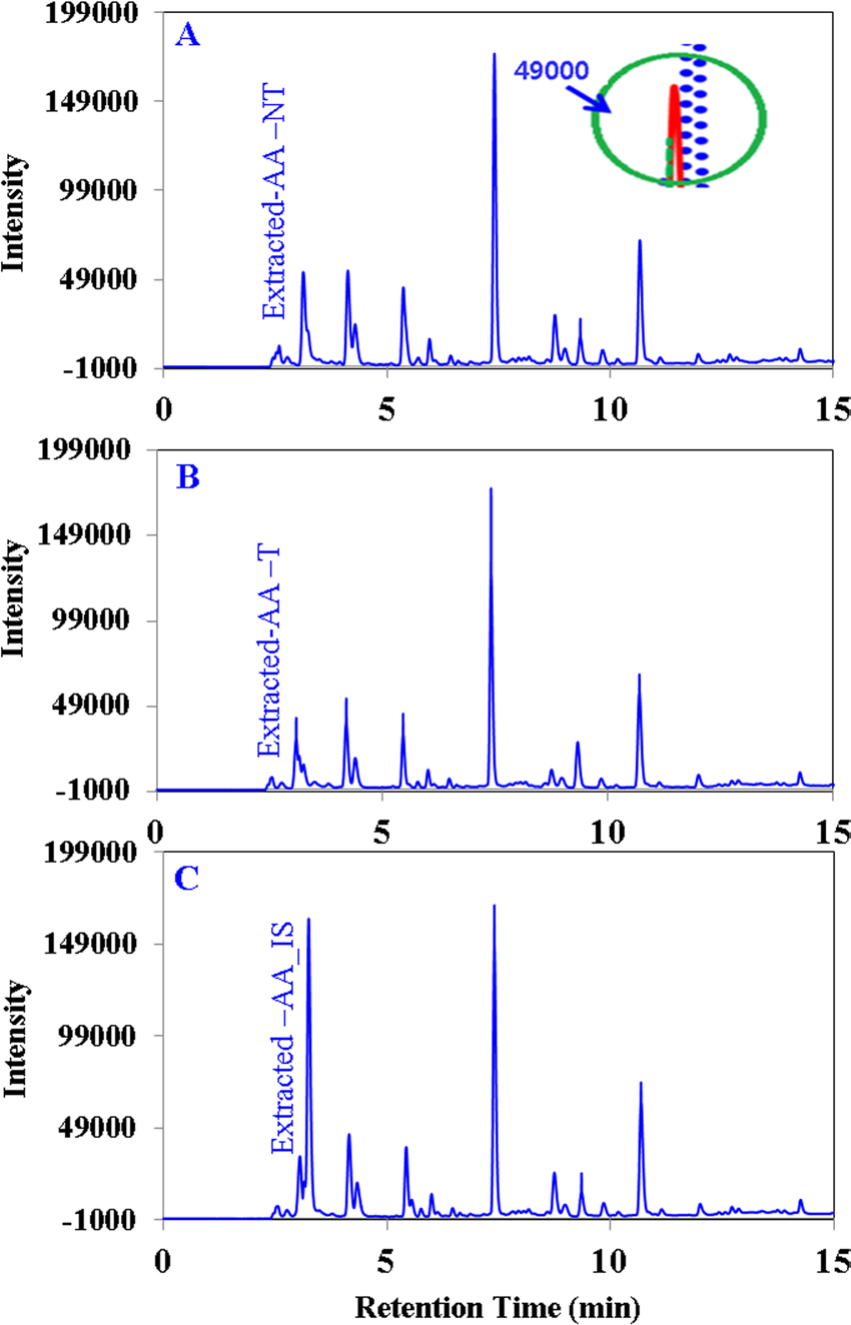
HPLC chromatograms (monitored at 280 nm) showing ascorbic acid extracted from onion slices. (**A**) Non-treated, (**B**) treated with air (**C**) with internal standard to ensure accurate identification of ascorbic acid. Inset: magnified superimposed peak view of ascorbic acid chromatograms at intensity 49000 to assess the reduction level of ascorbic acid easily.

Figure 3C shows that the ascorbic acid standard was nearly destroyed within 1 minute of plasma operation with clean dry air feed gas and no fragmented compounds were identified by our present setup a magnified view for the standard ascorbic acid obtained at 3.30 min was shown as an inset in Fig. 3C. In case of N_2_ feed gas 25% of standard ascorbic acid was destroyed within 10 min of plasma operation (Fig. 3D) and no fragmented compounds were identified a magnified view for the ascorbic acid standard obtained at 3.30 min was also shown as an inset in Fig. 3D. The amount of ascorbic acid loss in onion slices in 10 min of plasma treatment is shown in Table 1; treatment with clean dry air feed gas shows the higher reduction (11.82%) of ascorbic acid compared to treatment with N_2_ gas (7.98%). Figure 5A and B show chromatograms of extracted ascorbic acid before and after plasma treatment with clean dry air gas. Figure 5C shows the spectrum with the addition of an internal standard to ensure that the extracted product is the target analyte. Chromatograms obtained before and after treatment are similar in terms of the peak obtained at different retention times. The chromatogram for the N_2_ feed gas was not shown because the pattern obtained is somewhat similar to the pattern obtained for clean dry air gas and because the reduction of quercetin and ascorbic acid was also smaller. Blank experimental result confirms that 5.21% ascorbic acid reduction occurs due to leaching of water for the highest operation time (10 min) used in this experiment. No significant differences were observed for the alteration of the feed gas used.

### Evaluation of surface color parameters of onion slices

The changes in color parameters (L*, a*, b*) of onions treated with clean dry air and nitrogen gas were measured and shown in Table 2. Plasma treatment for 10 min resulted in ΔE* values of 1.03 and 1.90 for the clean dry air and N_2_ feed gas treatments, respectively. According to reference^27^, total color differences (ΔE*) are considered to be very distinct (ΔE* > 3), distinct (1.5 < ΔE* < 3), or small differences (ΔE* < 1.5). In our experiments, the ΔE* values are in the small difference range (ΔE* 1.03) for air treatment and in the distinct range (*ΔE* 1.90) for the N_2_ treatment. The leaching contributed more in color change as compare to plasma operation, whereas variation of feed gas does not contributed much. The overall color changes result is tabulated in the Table 2 instead of the detail for blank experiments results.

**Table 2:** The L*, a*, b*, and ΔE* values for onion slices after 10 min of plasma treatment for non-treated (Control), treated with clean dry air gas (Air-Plasma), and treated with N_2_ gas (N_2_-Plasma).

Treatment	L*	a*	b*	***Δ***E^2^	***Δ***E ± SD
Control	78.59	−3.40	13.98		
	78.64	−3.43	14.04		
	78.53	−3.37	13.97		
	78.67	−3.39	13.98		
	78.58	−3.42	14.03		
Air-Plasma	78.93	−3.04	13.15	0.93	1.03 ± 0.10
	78.90	−3.03	13.14	1.38	
	78.81	−3.01	13.13	0.91	
	78.70	−2.99	13.10	0.93	
	77.89	−2.98	13.12	1.50	
N_2_-Plasma	77.36	−2.96	12.66	3.45	1.90 ± 0.09
	77.25	−2.95	12.62	4.18	
	77.42	−2.98	12.64	3.15	
	77.3	−2.99	12.68	3.73	
	77.37	−2.96	12.68	3.50	

## Discussions

**Figure 6:**
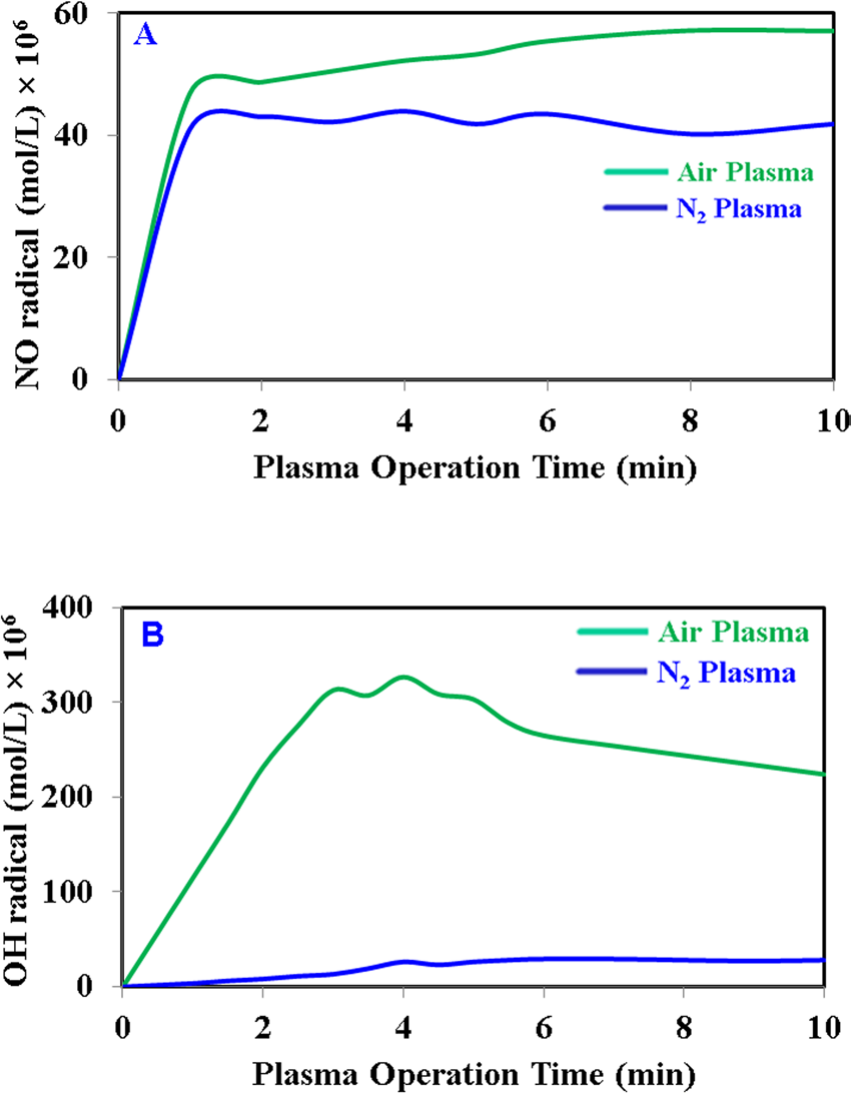
Measurement of radicals concentration (**A**) oxides of nitrogen (NO) and (**B**) hydroxyl radical (OH).

DBD plasma discharged water treatment was performed successfully to determine the efficiency and the mechanism of inoculated *S*. Typhimurium inactivation and the changes in physicochemical properties of onion. Among the radicals produced by DBD plasma discharged water, OH radicals play a major role in inactivation, with dissolved O_3_ gas ranking next in importance. H_2_O_2_ was found to play no role in *E. coli* O157:H7 inactivation in our study because the amount of hydrogen peroxide produced during our treatment time was very low (2.5 × 10^−6^ M) and remained constant throughout the operation^25^. The bactericidal effect of ozone is well known and has already been used for sterilization in various industries^28^. However, some researchers found that up to 28 ppm of ozone gas had no effect on *E. coli* O157:H7 inactivation when they performed a 30 min treatment with a DBD generator in room air at 60% relative humidity^29^. In our previous study it was observed that the reduction of planktonic *E. coli* O157:H7 cells by RNS were not very significant with this current plasma set up. Toshihiro *et al*., 2014 reported that N_2_ plasma can generate positive nitrogen (N^+4^) with three-body collisions, and underwater it produces NO_2_^−^, NO_3_^−^, NH_3_ and OH radicals^30^. The NO concentration obtained for both gases is somewhat similar shown in Fig. 6A, the highest concentration obtained for nitrogen oxides is about 5.8 × 10^−7^ M for air feed gas, hence, at this lower concentration, nitrogen oxides do not play a major role in bacterial inactivation. It was already shown that the majority of bacterial inactivation occurs by OH radical and O_3_^25^; therefore the lower inactivation obtained with N_2_ plasma is obvious as the measured OH radical (secondary species) concentration was also much lower (Fig. 6B) as compare to OH radical generated for air plasma. However, to understand the effect of nitrogen radicals and NH_3_, extensive research needs to be performed through some alternative approach, which is beyond the limit of the present study. Effect of UV was measured using a UV lamp for 10 min of exposure instead of plasma source and no inactivation was found for the UV generated by our present set-up (data is not shown here). From the above discussion, it is clear that DBD plasma discharged water significantly reduces *S*. Typhimurium at different rates for the different feed gases used. Clean dry air gas shows higher inactivation compared to N_2_ gas with 10 min of plasma operation. In this study, the concentration obtained for control sample is equal to the concentration of S. Typhimurium inoculated on onion slice (~10^6^ logCFU/mL), hence we can say there was no antimicrobial activity shown by onion slices during the entire experimental procedure. Also, the water temperature does not have any effect on microbial reduction as it remains constant throughout of the plasma operation.

In our previous work, it was observed that 1 min of DBD plasma discharged water treatment can reduce 2.5 log of *S*. Typhimurium from perilla leaf surface^31^. In this work, 2.5 log of inoculated *S*. Typhimurium reduction was obtained after 5 min of plasma treatment (Fig. 2). The differences in the inactivation efficiency may be attributed to differences in the surface roughness, thickness and moisture content of the tested samples. The slices were dried for one hour to let the surface liquid water evaporates and to settle the microbes on the surface; internal juices in the tissue continue to diffuse out. According to reference^32^, the more residual moisture remains on the slice, the easier it is for microbial cells to move and penetrate deep into the slice, which enhances the chances of their survival. Surface thickness and moisture content might play the major role in the lower inactivation efficiency when *S*. Typhimurium is inoculated on onion than on perilla leaf as both the surface thickness and moisture contents are higher for onion slices.

The efficiency of DBD plasma on *S*. Typhimurium inactivation in water was evaluated in our previous work^31^ (*E.-J. Lee et al*.^31^), it was observed that approximately 7.0 log of reductions occur within 80 sec and incase of pre-treated water complete inactivation occurs within 20 sec. This express reduction for bacterial suspension in water helps us to decide not to perform any post-treatment measurement in regards to the bacterial survival numbers in the leftover water of working vessel. However, observed 4% bacterial detachment during 10 min of plasma operation, result obtained from blank experiment, is considered to be the presence of very less number of S. Typhimurium in water. Therefore, with the evidence of our previous result we can infer that, the possibility of surviving of this fewer numbers of S. Typhimurium is almost impossible after 10 min of plasma treatment.

Flavonoids are known to be natural OH radical scavengers, and oxidative degradation of flavonoids occurs upon interaction with hydroxyl free radicals, leading to the formation of low molecular weight phenolics. The degradation follows pathways similar to heat-induced oxidative cleavage^23,33^. Thus, questions were raised concerning the plasma chemical interactions of flavonoids and how such interactions could result in flavonoid degradation. To elucidate the influence of plasma immanent species on stability, quercetin was chosen as one of the flavonoids present in onion. Figure 3A shows that quercetin was cleaved into lower molecular weight phenolic compounds when the clean dry air feed gas was used. The smaller fragments underwent some further chemical reactions with the plasma generated species to degrade or produce secondary fragments, as after 90 s (Inset of Fig. 3A) of plasma treatment the fragmented peaks started decreasing and by 10 min they disappeared. No lower molecular weight compounds were observed in the chromatogram (Fig. 3B) when N_2_ gas was used as a feed gas, most likely due to the different radicals generated by plasma producing different types of compounds for the different feed gases. Figure 3A and B also shows that quercetin degradation occurs at a faster rate with clean dry air feed gas compared to N_2_ gas. From the above discussion it is ascertained that the rate of degradation and the product generation for plasma inactivation depends on the feed gas used. Detailed mechanism elucidation of the reaction between plasma generated radicals and quercetin was not the primary focus of this study; hence, further experiments were not performed in this regard. However, when plasma was applied on onion slices, there were no such additional peaks observed in Fig. 4 before versus after plasma treatment for 10 min, which was observed to start appearing after 30 s of plasma treatment in the quercetin standard chromatogram (Fig. 3A). The absence of additional peaks on the extracted quercetin chromatogram (Fig. 4) after plasma treatment for 10 with respect to the chromatogram from before plasma treatment is the evidence that no quercetin degradation occurs by the generated radicals during plasma treatment. For clarity (generation of any lower molecular weight phenolic compounds), the quercetin content was also measured at different plasma treatment times such as 0.5, 1, 1.5, 3 and 5 min (data not shown here), and no additional peaks were observed (like Fig. 3A) with respect to the non-treated one. However, a small quercetin reduction was measured (3.74–5.07%, Table 1) for both the feed gases used, where leaching contributed minimum of 2.13%. If rest of the slight reduction measured in this study truly happen for the radicals generated, therefore we may consider the absence of the lower molecular weight fragmented peaks in the extracted quercetin chromatogram is the out of detection limit of HPLC. Hence, the causes of the small reductions were remaining unknown except leaching. Less penetration depth^22^ of the plasma species is advantageous for protecting the chemical contents, i.e., quercetin, of the onion, or the complex nature of the food item helps to protect its chemical nature against plasma radicals.

The effectiveness of plasma radicals at reducing ascorbic acid in onion slices was also found to be insignificant; the greatest loss of ascorbic acid, 11.82%, was obtained in the case of clean dry air feed gas, whereas N_2_ feed gas plasma reduces ascorbic acid by only 8.0%. Elez-martinez *et al*.^34^ studied the effect of pulsed electric field (PEF) treatment on vitamin C concentration in orange juice and found that a maximum 12.5% reduction occurs, which was lower than the reduction induced by pasteurization at 90 °C for 1 min (17.6%)^34^. Therefore, our result for ascorbic acid (vitamin C) reduction is considered to be within an acceptable range. Among the reduced ascorbic acid, at least 5.01% was contributed by leaching. The rest of the reduction of quercetin and ascorbic acid content that occurs especially on the surface of the onion slices is most likely due to oxidation by the generated plasma radicals^22^. In addition, ascorbic acid is light sensitive^35^; therefore, UV generated by the plasma may also play an important role in the higher ascorbic acid degradation compared to quercetin degradation. Using N_2_ gas provides lower reduction for both quercetin and ascorbic acid.

Changes in the color parameters (L*, a*, b*) of onion slices from DBD plasma discharged water treatment were measured, and little visible change was found to occur on the onion slices. The major changes occur due to leaching of water during the operation; rest of the slight changes may contributed by the radicals generated or some other unknown reasons. The redness (a*) for air plasma and N_2_ plasma treated onion increases slightly over that of the non-treated onion, whereas the yellowness (b*) decreases for both the cases. The lightness (L*) of the N_2_-treated onion decreases slightly, but that of the air-treated onion remains the same. Considering the overall results for color parameters, to protect the original color, using clean dry air gas is advantageous. As a whole it is clear that DBD plasma discharged water treatment shows significant effects on reducing inoculated *S*. Typhimurium, with different reduction levels for the different feed gases used. The color parameters, quercetin content and ascorbic acid content of the sliced onions were found to be only minimally affected, and the changes that occurred were measured to be in the acceptable range. Considering the data obtained for color parameters, quercetin content and ascorbic acid content of the sliced onions, clean dry air shows better performance than N_2_ as plasma feed gas. To the best of the authors’ knowledge, this study is one of the first attempts to provide a quantitative analysis of the effect of DBD plasma discharged water on the physiochemical properties of treated onion sample. The results presented here can be considered a successful use of DBD plasma discharged water treatment for bacterial inactivation of surface contaminated fresh vegetables. However, further studies are required for the potential use of DBD plasma discharged water treatment in any large-scale industrial application for food decontamination.

## Methods and Materials

### DBD plasma discharged water apparatus

A schematic diagram of the experimental apparatus and conditions used in this work is shown in our previous work, Khan *et al*. (AIP Adv. 2015)^25^. The DBD plasma discharge apparatus consists of an electrode (DBD reactor), a power supply, and a gas supply. A quartz tube with an internal diameter of 30 mm was used as a dielectric barrier. Two rod-type copper (Cu) electrodes that were 7 mm in diameter and a ground Cu electrode that was 1 mm in diameter were coiled around the quartz tube. The plasma discharge occurred between the tube and the ground electrode. A neon transformer (18 kV, 20 kHz) with an input voltage of 220 V was used as the power supply. To diffuse the generated plasma, feed gases were fed into the inside of the quartz tube at a rate of 5 L/min, with the flow rate controlled by a regulator. The DBD reactor was submerged in an acrylic water bath (450 × 200 × 155 mm^3^), with the water in the bath acting as a coolant. The plasma jet was diffused through a round shaped diffuser in a separate plastic water jar by immersing the round bubbler in 2.0 L of distilled water. The water temperature in the vessel was measured to be 22 °C and remains constant throughout the plasma operation.

### Descriptions of Feed gases

To evaluate the effect of feed gases on microbial reductions with DBD plasma discharged water two different feed gases i.e., clean dry air gas and pure N_2_ gases were purchased from a local distributor of Deokyang Co., Ltd, Ulsan, republic of Korea. The clean dry air gas composition was 21% of O_2_, 0.10 µmol/mol of CO, 1.90 µmol/mol of H_2_O, and the rest is N_2_. The purity for N_2_ gas was 99.999% with ≤0.04 µmol/mol of T.H.C., ≤3.0 µmol/mol of O_2_, ≤1.9 µmol/mol of H_2_O, ≤0.08 µmol/mol of CO_2_.

### Inoculation of Salmonella Typhimurium on onion and DBD plasma discharged water treatment

Onions were purchased from a local market and were sliced into 3 cm × 3 cm samples. To inoculate *S*. Typhimurium on the sliced onion, a 100-fold diluted (approx. 10^6^ CFU/100 ml) cell suspension was prepared from *S*. Typhimurium overnight culture, 100 µl aliquots of inoculum per slice was inoculated on the back side of the onion slices. The droplets from 100 µl aliquots were deposited on several different locations. Inoculated samples were dried for 1 h on a clean bench. Several slices of onion (approximately 20 g) were immersed in the water (2 L, 20 °C) that contained the round shaped diffuser. All samples were treated for 1, 3, 5 and 10 min with the plasma device switched on. After treatment, samples were immediately transferred to a sterile bag (B01196, Nasco Whirl-Pak®, Fort Atkinson, WI, USA) with D/E broth (D/E Neutralizing Broth w/Tween) and homogenized with a BagMixer® 400 (Interscience, Saint Nom, France). The enumeration of *S*. Typhimurium was carried out by spread-plating the homogenate, appropriately diluted in saline, on XLD agar (Xylose Lysine Desoxycholate Agar, Difco™) and incubated at 37 °C for 24 h. For statistical analysis, minimum three independent experiments were carried out for each treatment. A blank experiment was performed (negative control) to rule out the effects of water circulation on *S*. Typhimurium removal from the sliced onion surface in a 10-min treatment in the absence of plasma (with the plasma source turned off).

## Measurement of Chemical Properties

### Extraction and HPLC analysis of quercetin

To extract quercetin from onion, slices were homogenized and extracted using the conventional solvent extraction procedure explained in^36^. 2 g of onion mash was heated in a water bath with 10 ml of 80% methanol for 1 h and then filtered using normal filter paper and a hydrophilic membrane (0.45 *μ*m). The non-treated filtrate obtained and those treated with clean dry air gas and N_2_ gas were stored for HPLC analysis. The method used in this study is similar to the method described in Adam *et al*.^37^. The HPLC analyses were performed with a Luna 5U-C18 (2) 100 A column (250 mm × 4.5 mm, 5 µm) plus, equipped with a Jasco quaternary gradient pump (pu-2089) and a Jasco UV-2077 4λ intelligent UV/vis detector. The compounds were eluted with a gradient elution of mobile phases A and B. Solvent A consisted of deionized water and 1% acetic acid, and solvent B consisted of methanol (HPLC grade) and 1% acetic acid. Acetic acid (1%) was added to reduce peak tailing. The gradient elution program was modified from that described to obtain better separation at a faster rate. The program was as follows: 10.0% B–17.2% B (5 min) 17.2% B–23.0% B (10 min), 23.0% B isocratic (10 min), 23.0% B–31.3% B (5 min), 31.3% B–46.0% B (10 min), 46.0% B–55.0% B (5 min), 55.0% B–100% B (5 min), 100% B isocratic (10 min), 10.0% B (5 min) and 10.0% B isocratic (5 min). The injection volume for all samples were 50 µl. Quercetin was monitored at 280 nm at a flow rate of 1 ml/min. All determinations were performed in triplicate. In the case of quercetin, the treatment and measurements were performed at 0.5, 1, 1.5, 3, 5 and 10 min. Quercetin was identified by matching the retention time and spectral characteristics against those of standards, and the quercetin contents were determined using calibration curves. A blank experiment was performed (with the plasma source turned off) to investigate any quercetin reduction occurs due to the leaching of water during 10 min plasma operation.

### Extraction and measurement of ascorbic acid

We assessed the changes in ascorbic acid content caused by DBD plasma discharged water treatment with high performance liquid chromatography (HPLC). The method used in this study for extracting ascorbic acid is similar to the method described in reference^38^. The non-treated onion slices and those treated with clean dry air gas and N_2_ gas were blended into a homogenate, which was then filtered using normal filter paper and hydrophilic membrane (0.45 *μ*m). The extracted ascorbic acid was stored in a vial for HPLC analysis. For ascorbic acid analysis, several gradient conditions were changed to obtain a better separation at faster rate; the program was as follows: 25.0% B–50.0% B (2 min) 50.0% B isocratic (3 min), 50.0% B–70.0% B (3 min), 70.0% B–100.0% B (2 min), 100% B isocratic (2 min), 50.0% B (3 min) and 10.0% B isocratic (5 min). Ascorbic acid was monitored at 280 nm at a flow rate of 1 ml/min. All determinations were performed in triplicate. The treatment and measurements were performed at 0.5, 1, 1.5, 3, 5 and 10 min. The concentration of ascorbic acid was measured by HPLC and calculated by a standard calibration method. Ascorbic acid was identified by matching the retention time and spectral characteristics against those of standards, and the content was determined using calibration curves. A blank experiment was performed (with the plasma source turned off) to investigate any ascorbic acid reduction occurs due to the leaching of water during 10 min plasma operation.

### Measurement of color parameters

**T**o quantify the color differences in the onion slices, the slices were homogenized and examined with a colorimeter (Minolta CM-700d spectrophotometer, Konica Minolta Optics, Inc., Osaka, Japan) calibrated to a standard white tile (L* = 93.52, a* = 0.32, b* = 0.33). The color was measured for non-treated and plasma-treated (10 min) homogenized onion. The L*-axis represents the degree of brightness within a sample, ranging from 0 (black) to 100 (white). The a* axis denotes the degree of green (−)/red (+), while the b* axis represents the degree of blue (−)/yellow (+) in the sample. *Δ* denotes the value difference before and after the treatment in each group. The total color difference (ΔE*) of the specimen can be calculated from the formula (ΔE* = [ΔL*^2^ + Δa*^2^ + Δb*^2^]^**1/2**^)^39^. Homogenized onions were placed inside a Petri dish and placed in front of the camera of the colorimeter, and the L*, a*, b* values were determined, from which ΔE* was calculated. All determinations were performed in triplicate. The treatment and measurements were performed at 0.5, 1, 1.5, 3, 5 and 10 min but the data shown only for the 10 min measurements. Two blank experiments were performed (with the plasma source turned off) for the two different feed gases to investigate any color changes occur due to the leaching of water during 10 min plasma operation.

### Optical emission spectroscopy (OES)

OES was applied to identify some of the active species in the plasma. End-on light emission was collected via a fiber optic cable and imaged to the entrance slit of a 0.75-m spectrometer (Princeton Instrument/Acton Spectra Pro 2750) equipped with an 1800-groove/mm blazed holographic grating. To obtain a reasonable signal-to-noise ratio with sufficient spectral resolution to isolate the major emission lines, the entrance slit was set to 100 *μ*m. An intensified CCD camera (Princeton Instrument I-Max-512) was used to record the dispersed emission spectra.

### NO radical measurement

Nitric oxide (NO) concentration in the water for both air and nitrogen feed gases were determined using a NO assay kit QuantiChromTM Nitric Oxide Assay Kit, (D2NO-100) after 8 min of the starting of plasma device. The optical density was read at 540 nm using a Quant 2000 spectrophotometer and the concentration was measured with standard calibration method.

### OH radical measurement

The concentrations of OH radical generated for both air and nitrogen feed gases were measured using terephthalic acid (TA, Sigma Aldrich, USA). TA was dissolved in 0.5 M of NaOH solution to prepare 2 × 10^−2^ M of TA solution; NaOH solution was used because TA does not dissolve in a neutral or acidic medium. The pH of the TA solution was 9.73. When the solution containing TA and 2-hydroxyterephthalic acid (the product of TA and OH radical, HTA, Sigma Aldrich, USA) molecules is irradiated by UV light (λ = 310 nm), HTA molecules emit light at λ = 425 nm, while TA molecules do not. The fluorescence intensity of HTA is independent of pH in the 6–11 range. A multi-mode micro plate reader system (SpectraMax i3- Molecular Device) was used for the HTA fluorescence measurement. A fluorimeter was used to measure the intensity of HTA, and the concentration of 2-hydroxyterephthalic acid was used with a standard calibration method. The stoichiometry of the reaction between the OH radical and TA was used to calculate the concentration and the rate of OH radical generation at a particular time. The detailed procedure for measuring the OH radical concentration will be described elsewhere^25^.

### Statistical analysis

The data were analyzed via SPSS statistical program (IBM-SPSS 22.0.0.0), and the significant differences were compared with untreated control, Mann–Whitney-U (p < 0.05) a non-parametric test. The experiments were repeated a minimum of three times unless stated otherwise.
