# Publisher Correction: Feed gas effect on plasma inactivation mechanism of *Salmonella* Typhimurium in onion and quality assessment of the treated sample

**DOI:** 10.1038/s41598-018-24190-9

**Published:** 2018-04-13

**Authors:** Muhammad Saiful Islam Khan, Eun-Jung Lee, Seok-In Hong, Yun-Ji Kim

**Affiliations:** 1 Department of Food Biotechnology, University of Science and Technology, Daejeon, 305-350 Republic of Korea; 2 Faculty of Food Science and Biotechnology, College of Life Science, Sejong University, 209 Neungdong-ro, Gwangjin-gu, Seoul 05006 Republic of Korea; 3 Food Safety Research Group, Korea Food Research Institute, 245 Nongsaengmyeong-Ro Iseo-Myeon, Wanju-Gun, Jeollabuk-Do 55365 Republic of Korea

Correction to: *Scientific Reports* 10.1038/s41598-017-17579-5, published online 18 December 2017

The original version of this Article contained a typographical error in the spelling of the author Seok-In Hong, which was incorrectly given as Suk-In Hong. This has now been corrected in the PDF and HTML versions of the Article.

